# Gaze behaviour to lateral face stimuli in infants who do and do not receive an ASD diagnosis

**DOI:** 10.1038/s41598-020-69898-9

**Published:** 2020-08-06

**Authors:** Georgina Donati, Rachael Davis, Gillian S. Forrester

**Affiliations:** 1 Department of Psychological Sciences, School of Science, Birkbeck, University of London, Malet Street, London, WC1E 7HX UK; 2 Psychology Department, University of Edinburgh, Edinburgh, EC1V 0HB UK

**Keywords:** Evolutionary theory, Evolutionary developmental biology, Cognitive neuroscience

## Abstract

Cerebral lateralisation of function is a common characteristic across vertebrate species and is positively associated with fitness of the organism, in humans we hypothesise that it is associated with *cognitive* fitness. This investigation evaluated the early development of lateralised gaze behaviour for face stimuli in infants at high and low risk for autism from the British Autism Sibling Infant Study (BASIS). The BASIS cohort includes a low risk group and three high-risk groups who at age 3 were developing (i) typically, (ii) atypically or (iii) had received a diagnosis for ASD. Using eye-tracking data derived from a face pop-out task at 6 and 14 months of age, all non-ASD groups showed a bias for stimuli on the left at both timepoints. At 6 months the ASD group demonstrated a preference for stimuli on the right and were slower than their neurotypical counterparts to look at faces on the left. However, by 14 months these differences disappear. Longitudinal associations between lateral looking behaviour at 6 months and language and motor ability at 14 months were also found. Results suggest that infants who go on to be diagnosed with autism exhibit early differences in gaze behaviour that may be associated with subsequent cognitive outcomes.

## Introduction

For all animals, motor actions are performed to facilitate behaviours that are biologically adaptive^1^. For many vertebrate species, these behaviours are often found to be dominantly produced on one side of the body, resulting from ‘*cerebral lateralisation’*. Cerebral lateralisation is the asymmetric contributions of the two hemispheres of the brain for interacting with the environment^2,3^. Patterns of side-biased visual and motor behaviours are common to a large range of vertebrate species (e.g.^3^) suggesting that throughout evolutionary time, the right hemisphere emerged as dominant for responses to danger and novelty in the environment (e.g. predators), while the left hemisphere became dominant for producing motor responses that require routine and structured sequencing (e.g. feeding) (for a review see^4^).

In modern humans, lateralised sensory and motor functions are core components of mental processing^5^ and support the emergence of higher cognitive functions. The left hemisphere dominance for structured motor sequences supports the fine articulation behaviours underpinning tool use (such as writing) and speech^6^ resulting in population-level right-handedness^7^ and left hemisphere language dominance. The right hemisphere’s dominance to perceive and respond to environmental threats^8^, supports modern human social-emotional-processing^9^.

A common measure of lateralised processing uses visual field biases. In animal species with forward facing eyes and binocular vision, like humans, both eyes have a left and a right visual field that is processed by the opposite hemisphere. Humans demonstrate a robust left visual field (LVF) bias for perceiving human faces^10–12^, and for detecting emotional expressions^13,14^. This means they will look more to the right side of the face. Moreover, in laboratory studies where participants are presented with the same face stimuli to both visual fields, they report those in the LVF to be more salient than those presented to the right visual field^15^. Neurotypical adults showed an increased number of first saccade fixations to the left of faces during gender identification^16^. Studies using fMRI have consistently shown increased right hemisphere activation, for example, in fusiform gyrus, when viewing faces (for review, see^17^) and facial expression affect valence^18^. fMRI and eye-tracking data have indicated that the degree of an individual’s LVF bias is positively associated with the right hemisphere’s lateralised activity level when processing face stimuli. Moreover, longitudinal analyses suggest that an individual’s level of lateralisation appears stable over time^19^. Thus, the dominant hypothesis for the LVF advantage is due to a right hemisphere (RH) specialisation for face processing abilities in the majority of individuals. This pattern of visual biases is not unique to humans. It is also visible also in other vertebrate species^20,21^.

Evolutionary theories suggest that cerebral dominances for basic survival behaviours have provided a foundation for the emergence of human higher cognitive functions (e.g. ^9,22^). Although ontogeny does not recapitulate phylogeny in the literal sense^23^, during human cognitive development, higher processes are supported by and scaffold atop of early perceptual and motor capabilities^24^, which are, by evolutionary design, cerebrally lateralised. Therefore an ‘evo-devo’ theory would predict that early markers of cerebral lateralisation such as contralateral motor biases would appear early in development and predict later cognitive development. Furthermore, it has been argued that typical cognitive development results from the clear separation of hemispheric dominances associated with strong behavioural biases^25^ and that weak or absent cerebral dominances predict weaker motor biases which in turn are associated with poorer subsequent cognitive abilities^9,26–28^.

In typically developing children, a left-biased functional motor circuit connectivity is evident by 12 months of age^29^. Although, hand dominance can be predicted with 80% accuracy via foetus thumb-sucking behaviour in utero^30^. Only a handful of investigations have considered associations between motor biases and cognitive ability in developing children. For example, stronger motor laterality as measured by hand skill is related to greater cognitive ability^31^. The early development of strong handedness has been linked with typical language acquisition^32,33^ and increased vocabulary size^34^. Conversely, reduced right-handedness in young children has been associated with weaker language development (e.g.^35^). Motor lateralisation has also been associated with executive function (see^22^ for a review).

There is very little research looking at the developmental trajectory of right hemisphere social dominances and the LVF. However, two studies have found a LVF preference for faces in infants as young as 3 months^36,37^ where others have found it to emerge around 6 months^38^. Although, only one study has compared face and non-face stimuli in order to discriminate between a general visuo-spatial effect and a specific face preference^39^. Guo and colleagues found a LVF bias in infants of 6 months, but not a face-specific effect concluding that at 6 months this LVF preference is not yet specialised to faces^39^. Therefore, the typical development trajectory of this LVF bias for faces is still broadly unknown. Furthermore, these studies all use a single face presented centrally in the screen and look at the amount of time spent attending to the left vs the right side of the face. Some use naturalistic faces potentially confounding emotional expression with processing as there is evidence for asymmetries in the production of expressions on different sides of the face^10^. Furthermore, because few use a non-face control, it is not clear whether this is a face-specific effect. However other paradigms suggest this is the case—for example when navigating physically around a person or object, children from 5–14 years of age show a significant preference for choosing a rightward path for people, keeping the person in their left visual field, but no preference for objects^40^. Furthermore, children aged 4-5yrs have a preference for cradling a baby to the left, but no such preference for a weight, shape and visually matched sack^26^. Finally, a recent EEG study in newborn infants (1–4 days old) using face and non-face control stimuli found a face specific right lateralised neural response pattern. This suggests that early infant face processing may be dominant in the right hemisphere even from birth^41^.

Although atypical patterns of lateralisation are not necessarily a marker of pathology, weaknesses and/or reversals in typical patterns have been associated with atypical behaviour (e.g.,^42–44^). A growing body of evidence suggests that the typical pattern of cerebral lateralisation is not found in individuals with autism spectrum disorders (ASD), contributing to reduced social and communication abilities that commonly accompany an autism diagnosis. Individuals with ASD have shown anatomical disruption to the typically left lateralised language structures and atypically lateralised functional activation of these areas during linguistic tasks (see for a review,^29^). Compared with their neuro-typical counterparts, children with autism demonstrated a rightward shift in lateralisation for motor processes that was linked with decreased performance on several measures of the Physical and Neurological Examination of Subtle Signs (PANESS), suggesting an association between atypical motor lateralisation and motor performance in children with autism^45^. Moreover, instances of mixed-handedness appear to rise within populations of individuals with autism (17–47%) compared to that of the typically developed population (3–4%) (for a review see^46^). In terms of lateral gaze behaviour, children with ASD do not demonstrate the left cradling bias found in typical populations. These individuals revealed no lateral cradling bias, indicating a weakened or perhaps reversed laterality^47^ and cradling to the left in the typical population was associated with better social and communication scores^26^. A study by Dundas et al.^48^, assessed the LVF bias in infants at low and high risk of autism. The infants were assessed on how long they looked at the left vs right side of the face presented centrally. Here there was no LVF bias at 6 months in either group, but longer looking times to the left of the face appeared in the low risk group and not the high risk group by 11months^48^. Therefore, understanding a typical development of this left visual field specialisation process may help us understand any atypicalities that emerge during infancy.

Early biases in visual behaviour may act as a behavioural biomarker of typical and atypical patterns of cerebral lateralisation. However, it is still unclear when a LVF bias emerges in development, when it becomes specialised for faces and whether these lateralised behaviours are different in those with go on to be diagnosed with autism.

This prospective longitudinal investigation sought to reveal patterns of lateral gaze behaviour to face and non-face stimuli in infants at Low Risk (LR) and High-Risk (HR) for autism. Experimental measures were taken at 6 and 14 months of age and developmental outcomes were assessed at three years of age. This resulted in four experimental groups: LR typical; HR typical; HR atypical and HR ASD. Additionally we aimed to identify relationships between lateralised gaze behaviour at 6 months of age and subsequent cognitive ability at 14 months of age. Previous studies using this sample did not find any group differences in frequency of looking at faces and the current study seeks to establish whether these differences can be found in lateralised looking behaviour.

## Results

### Observations

**Figure 1:**
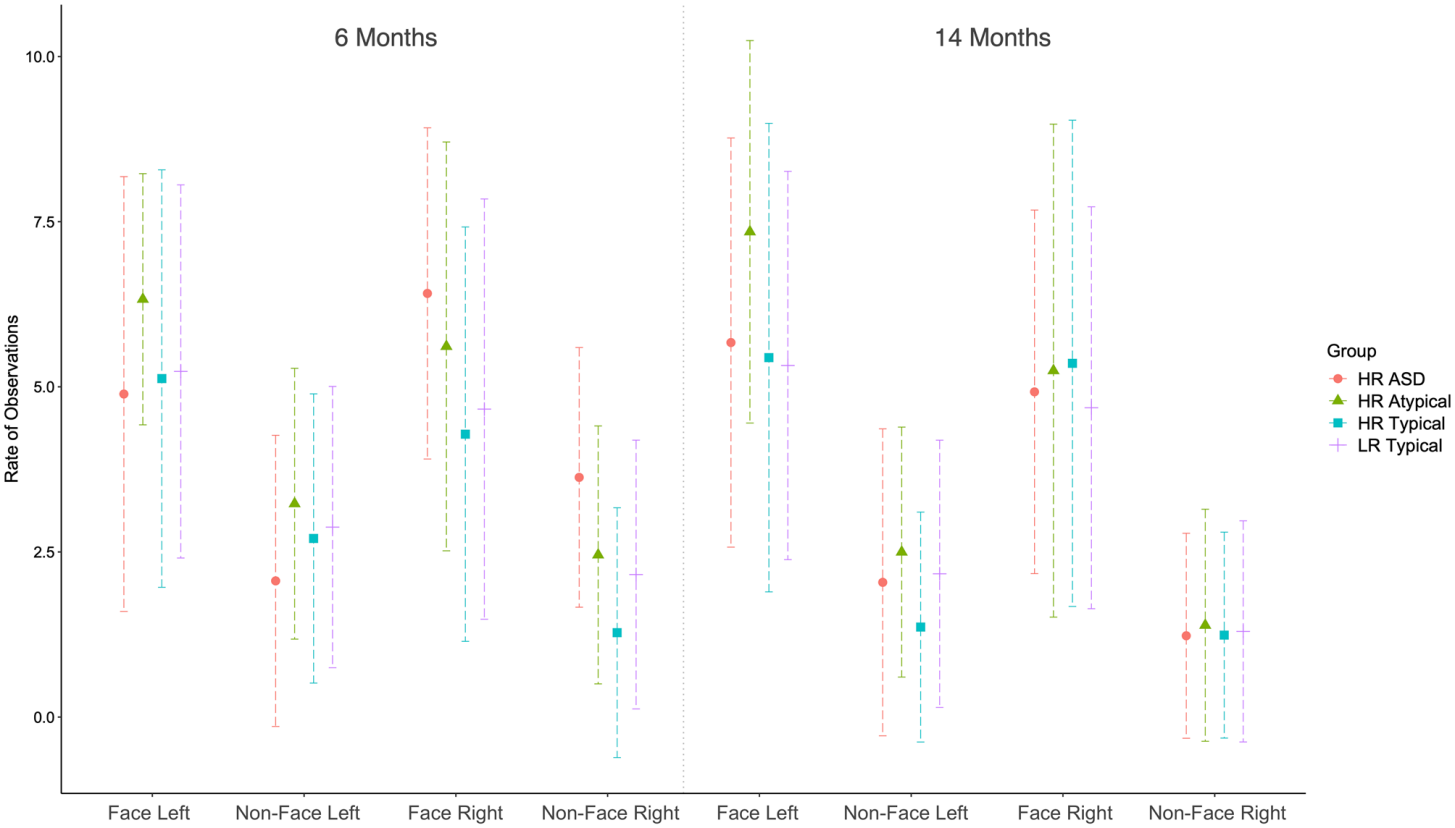
Number of observations looking left and right at faces and non-faces at 6 and 14 months of age for each of the four groups. Each line shows the findings for each group. Standard deviations can be found in Table 1.

At 6 months a significant main effect of Object was found (F(1, 396) = 109.027, p < 0.001, η^2^ = 0.216) and an interaction between Side and Outcome (F(3, 396) = 4.133, p = 0.007 η^2^ = 0.030) which was driven by the HR ASD group looking right more often in comparison to both the LR typical (p = 0.036) and HR typical groups (p = 0.003). At 14 months again a significant main effect of Object was found (F(1, 396) = 204.672, p < 0.001, η^2^ = 0.341), as well as a main effect of Side (F(1, 396) = 7.654, p = 0.006, η^2^ = 0.002) (Fig. 1). Results showed the same patterns in non-parametric tests.

### Speed of looking

**Figure 2:**
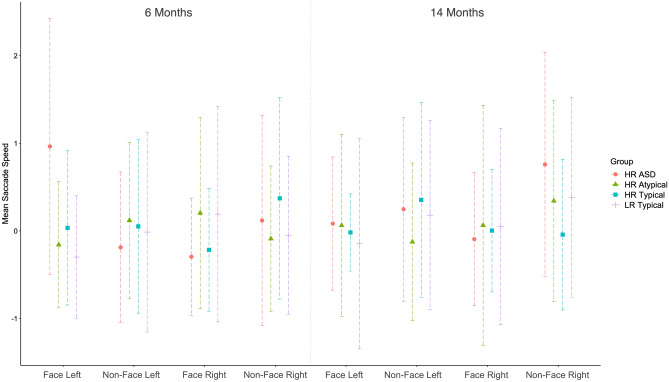
Saccade speed for looking left and right at faces and non-faces. Graphs created with the pooled means of all imputed datasets. Each line shows the findings for each group. Pooled standard deviations can be found in Table 1.

A significant 3-way interaction between Side, Object and Outcome (F(3, 1767.81) = 2.919, p = 0.033) was found at 6 months. Post-hoc tests suggest this effect was almost entirely driven by differences in the LR typical vs HR ASD group and mostly in the left face condition (16/50 imputation post-hoc analyses p = 0.01–0.05) where individuals with HR ASD were on average slower at looking at face on the left (Fig. 2). Complete case analysis for speed (N = 25) found the same pattern of results (Side × Object × Outcome at 6 months = N = 25, F(3, 181) = 3.978, p = 0.009). No effects were significant at 14 months for speed of looking in the imputed or complete case (N = 16) analyses.

Turning to the multiple regressions, fine motor ability at 14 months was not predicted by any observation measures, gross motor at 14 months was positively associated with number of face observations to the left at 6 months (*ß* = 0.26, p = 0.026). Receptive language was negatively associated with number of observations to non-faces on the right (*ß* = −0.27, p = 0.016) as was expressive language (*ß* = -0.25, p = 0.027). Multiple regressions using the 6 months speed variables as predictors did not explain any variance at 14 months in any of the language or motor scores. Full results can be found in Supplementary Table 1.

**Table 1:** Descriptive Statistics of the eight measures created from the Face Pop-Out task and used in the subsequent analysis.

			LR typical Mean (SD)	HR typical Mean (SD)	HR atypical Mean (SD)	HR ASD Mean (SD)
**Number of observations**						
Observation Scores^a^	6 months	Left face	5.23 (2.82)	5.13 (3.16)	6.33 (1.90)	4.89 (3.29)
		Right face	4.66 (3.18)	4.28 (3.14)	5.61 (3.09)	6.41 (2.51)
		Left non-face	2.88 (2.13)	2.70 (2.19)	3.23 (2.05)	2.06 (2.20)
		Right non-face	2.16 (2.03)	1.28 (1.89)	2.46 (1.95)	3.63 (1.97)
	14 months	Left face	5.32 (2.94)	5.44 (3.55)	7.35 (2.90)	5.67 (3.10)
		Right face	4.68 (3.04)	5.36 (3.68)	5.25 (3.73)	4.92 (2.75)
		Left non-face	2.17 (2.02)	1.36 (1.74)	2.50 (1.89)	2.04 (2.32)
		Right non-face	1.30 (1.67)	1.24 (1.56)	1.39 (1.76)	1.23 (1.55)
**Speed of looking**						
Mean saccade scores^b^	6 months	Left face	− 0.30 (0.70)	0.04 (0.88)	− 0.16 (0.72)	0.97 (1.46)
		Right face	0.19 (1.23)	− 0.22 (0.70)	0.20 (1.09)	− 0.30 (0.67)
		Left non-face	− 0.01 (1.14)	0.05 (0.99)	0.12 (0.89)	− 0.19 (0.86)
		Right non-face	− 0.05 (0.90)	0.37 (1.15)	− 0.09 (0.83)	0.12 (1.20)
	14 months	Left face	− 0.15 (1.20)	− 0.02 (0.44)	0.06 (1.04)	0.08 (0.76)
		Right face	0.05 (1.12)	0.00 (0.70)	0.06 (1.37)	− 0.09 (0.76)
		Left non-face	0.18 (1.08)	0.35 (1.11)	− 0.13 (0.90)	0.25 (1.05)
		Right non-face	0.38 (1.14)	− 0.04 (0.86)	0.34 (1.15)	0.76 (1.28)

^a^These are rate scores (100/possible trials*observed trials) and then a square root transformation is applied. ^b^ These scores have been standardised. LR = low risk, HR = high risk.

## Discussion

In our ancient vertebrate ancestors, a division of labour between the two hemispheres may have allowed for the simultaneous execution of basic survival strategies. Today, these biases can also serve as informative behavioural markers of brain organisation (e.g. ^4^) and afford insight into how foundational motor and sensory systems evolved to support the development of higher cognitive processes. This study assessed the development of a left visual field bias for faces versus non-faces in typical and atypical populations and results suggest that faces presented to the left and right visual fields may be treated differently by individuals that go on to develop ASD compared with their neurotypical counterparts.

This study had three main findings at 6 months: a main effect of Object (faces), a Side × Outcome interaction and a Side × Object × Outcome interaction. In terms of observations, a marked difference in the rate of looking at faces vs looking at non-faces in all groups was found supporting previous literature showing that infants have a preference for looking at faces over other objects^49–51^. There was also a Side × Outcome interaction driven by the HR ASD group looking right more often than looking left, and in particular in comparison to the HR atypical group who had the highest proportion of looking left, and with the LR typical group. This tendency to look right more than left was the opposite pattern to that seen in all of the other three groups who looked left more, and to what would be expected by a general attentional bias. It is also interesting that the high-risk group who go on to develop atypically but not with ASD looked the most to the left overall which could possibly suggest some sort of compensatory behaviour. Looking more to the left is considered to be due to visuo-spatial attentional ability dominant in the right hemisphere^52^, looking at objects and faces more to the right could indicate a reversed or reduced laterality of attention or that at 6 months attention is being driven by other factors. This reversed laterality in ASD has been found in previous work in different modalities. For example, an fMRI study looking at the laterality of the motor circuit found that with children with ASD had stronger right-ward lateralisation of the motor circuit compared to typically developing children^45^.

There was no Side × Object interaction with number of observations, but this is not surprising since the task did not require infants to choose between looking at faces on the left or right and therefore preference for looking at faces over other objects, superseded any lateral differences. However, when looking at saccade speed, it was possible to assess these potential differences. Here a Side × Object × Outcome interaction was found, driven by the HR ASD group being significantly slower to look at faces on the left. Therefore, no lateral effect for the other groups was found, but only for the HR ASD group and in the opposite direction to that expected in typical development. This was specific to faces in the left visual field and therefore did not seem to reflect a general attentional difference.

At 14 months in terms of number of observations, a main effect of Side appeared, and a main effect of Object continued. This main effect of Side was driven largely by the HR atypical group who continued to look left more than the other groups. Previous studies in this sample have shown an equally strong orientating response to faces in children with and without ASD at 6 months—however, high risk infants spent more time looking at faces^49^. But by 3 years of age, those in the high-risk group had a reduced ability to recognise unfamiliar faces and longer looking at 6 months was associated with decreased face recognition ability at 3 years^53^. This suggests that increased looking behaviour is not always indicative of better future performance. In the current study, interestingly the differences seen in the HR ASD group (at 6 months) looking more to the right disappeared by 14 months and was replaced by the same pattern of looking as the other groups. This could be due to a delay in development or the emergence of compensatory mechanisms. Furthermore, in terms of saccade speed, no effects were found at 14 months suggesting that if the differences in the HR ASD groups observed at 6 months are genuine—they may have a particular developmental window in which they operate. Overall, the current study suggests that the LVF bias to faces, is still not present by 14 months in any of the populations tested. However, this finding may also be as a result of the paradigm which does not force an individual to choose between looking at faces on the left or right. Future studies could address this issue by presenting both on the left and right simultaneously: faces with faces; objects with faces; and objects with objects. However, we find no advantage in saccade speed for looking left at faces. It is possible that we find different results to previous work because the paradigm requires spontaneous orientating in the context of an array. The array introduces multiple distractor items not present in other paradigms. In each slide there are four possible items to look at and only one face potentially slowing saccade speeds. However, there may be advantages to having multiple images in that it is potentially more ecologically valid.

Regression analyses using measures at 6 months to predict motor and language outcomes at 14 months showed a positive relationship between number of face observations on the left and subsequent gross motor ability. Both receptive and expressive language ability were also negatively associated with the number of observations to non-faces on the right. These longitudinal associations between side gaze behaviour and motor and language ability could reflect a generalized reduction in hemispheric specialisation or cascading consequences of early differences in motor lateralisation. If it indeed is the case that more complex cognitive abilities, scaffold on more basic motor abilities, early differences in foundational social and motor cues could go on to impact later cognitive development. Specifically, a reduction in lateralised attention to faces could negatively impact all cognitive processes that build upon the interaction with social stimuli, which could include anything from recognising faces to building the mutual gaze and joint attention skills that precede language development. In order to build on these hypotheses, future studies would benefit from recording both lateralised social and motor functions, such as handedness, as well as their later proposed cognitive counterparts.

The sample sizes for the study were small even after imputation and therefore further research with larger samples would be necessary to draw any convincing conclusions. Nevertheless, the overall findings of this study suggest that it may be possible to discern differences in social processing infants who go on to develop ASD, from as young as 6 months of age. A slower response to faces in the LVF suggests early differences in functional specialisation, which potentially go on to influence other cognitive and motor abilities is present and visible early in development.

Revealing the associations between behavioural and brain asymmetries and their links with cognitive processes has the potential to yield substantial advances in the area of developmental psychology and bring about new and early clinical diagnostic practices and therapeutic interventions. The presence of these ancient functional and organisational brain traits, in humans, offers a unique opportunity to investigate the development of cognitive processes within an evolutionary framework. The ability to comprehend social signals is fundamental for the survival of all animals because they provide important information about how to respond to future events^54^. To date, little is known about the associations between left visual field dominance for social stimuli and social cognitive abilities in young children and infants.

## Methods

### Participants

Participants for this study come from the British Autism Sibling Infant Study (BASIS). BASIS is a UK-based network of collaborators facilitating autism research in at-risk infants (www.basisnetwork.org/). Families enrol their infants before the age of 5 months and attend multiple research visits at regular intervals until their child reaches three years of age or beyond. For the current study, 103 infants from BASIS participated (53 at risk (21 male), and 50 low-risk (21 male). Infants participated in the eye tracking task and Mullen assessments at the Centre for Brain and Cognitive Development at ages 6–10-months and 12–15 months. Infants who at-risk for developing autism were clinically assessed around the time of their third birthday (mean = 37.7 months, sd = 3.0) at the Centre for Research in Autism and Education, Institute of Education by an independent team. Assessments indicated that there were three sub-groups of infants: Typically developing; Atypically developing not ASD and ASD.

All methods were performed in accordance with the relevant guidelines and regulations of the 1964 Declaration of Helsinki. Ethical approval for the current study was made available through BASIS (NHS NRES London REC 08/H0718/76). Parents and/or legal guardians gave informed consent through BASIS, the authors had no access to any participant identifying information. Data can be requested at https://www.basisnetwork.org/collaboration-and-project-affiliation/ and full details can be found in Supplementary Note 1.

### Task

The eye-tracking face pop-out paradigm used in this study has been thoroughly described in^49^. Briefly, overall there were 14 trials, each trial presented five stimuli in a circle, two to the left, two to the right and one above or below a central fixation. The stimuli always included one face and four non-social stimuli: a car, a phone, a bird, and a ‘face noise’ stimulus (see^49^). Central stimuli and ‘face noise’ stimuli were removed from the analysis leaving only lateral stimuli of the 14 trials, five of had a face on the left and six had a face on the right. All the other lateral stimuli were coded as ‘Non-face’ stimuli. Individual’s trials were excluded if infants did not look first at central fixation and then to the left or right, or if the saccade was slower than 1000 ms or faster than 100 ms. The analysis makes the presumption that if infants first fixated on the central cue that stimuli on the left side of the circle was presented to the left side of space (preferentially engaging the left visual field) and stimuli on the right side of the circle was presented to the right side of space (preferentially engaging the right visual field).

### Measures

Four observation variables were created *Face Left Obs*, *Non-Face Left Obs*, *Face Right Obs* and *Non-Face Right Obs*. Due to the different rates of image presentation (i.e. it was always possible to see a lateral non-face, but only 5 with face left and 6 with face right) the number of trials the individual looked to the left or right at a face or non-face was first summed and then turned into a percentage of possible trials (100/possible trials*observed trials) (Table 1). A second set of variables were created which calculated the speed of looking to faces and non-face on the left and right. Speed of looking (saccade) was calculated by subtracting the time of central fixation from the time of the first saccade. Mean saccadic times in milliseconds were then calculated across trials so that each participant had a mean saccadic time for *Mean Face Left*, *Mean Non-Face Left*, *Mean Face Right* and *Mean Non-Face Right.*

The Mullen Scales of Early Learning (MSEL) were assessed in the infants at 14 months^55^. MSEL is a direct assessment of verbal and non-verbal abilities relevant from birth to 6 years across four domains—Visual Reception, Fine (M = 57.87, range = 20–80) and Gross Motor (M = 48.54, range = 20–80), Receptive (M = 44.66, range = 20–80) and Expressive (M = 46.16, range = 24–76) Language. This study used the Motor and Language scales assessed at 14 months of age with the prediction that early typical lateralisation measures would predict later motor and language ability.

### Analysis

Two 2 × 2 × 4 ANOVAs were performed for Side (left, right) × stimuli (face, non-face) × Outcome for number of observations at 6 months and 14 months (N = 103: LR typical = 50; HR typical = 24; HR atypical = 12; and HR ASD = 17). Observation measures were not normally distributed and so were square root transformed and the results from parametric tests were validated in non-parametric tests with untransformed data.

Two 2 × 2 × 4 ANOVAs were also performed for Side (left, right) × stimuli (face, non-face) × Outcome for speed of looking at 6 months and 14 months. The study design meant that infants had the option of looking left or right in each trial at different stimuli, this means that there were infants who never looked at a face or object on the left or right. The proportion of missing data was higher for non-face stimuli compared with face stimuli and also for stimuli appearing on the right versus on the left side of the experimental screen for the non-ASD groups. The HR ASD group on the other hand had more missing data for stimuli appearing on the left at 6 months but not 14 months (Table 2). However, there were no significant differences in the overall amount of missing data between groups and no correlations between the proportion of missing data and the variables measured (i.e. speed of looking left and right) or any of the outcome measures (Mullen scores).

**Table 2:** Proportion of missing saccade time scores relative to sample size in each variable for the entire sample at 6 months and 14 months.

	LR typical %		HR typical %		HR atypical %		HR ASD %	
	6 m	14 m	6 m	14 m	6 m	14 m	6 m	14 m
Left face	18.2	18.0	20.8	25.0	0.0	8.3	23.5	17.6
Right face	27.3	22.0	29.2	25.0	16.7	25.0	5.9	17.6
Left non-face	27.3	42.0	29.2	58.3	25.0	33.3	47.1	52.9
Right non-face	40.9	60.0	62.5	58.3	33.3	58.3	17.6	58.8

Darker cells indicate a higher percentage of trials.

LR = low risk, HR = high risk.

Therefore, to reduce bias in the saccade time analysis missing data, at both 6 and 14 months, was imputed for individuals with a maximum of two missing scores (> 75% of their observed data). Missing scores were imputed with Multiple Imputation using Chained Eqs. ^56^ in R^57^ using 50 imputation datasets and 100 iterations. Observation measures and Mullen scores were included in the imputation to improve the imputation model^56,58^. Analyses with these measures were performed using pooled imputation datasets which adjusts test statistics to account for the uncertainty in the missing data. This provided an overall N = 60: LR typical = 30; HR typical = 9; HR atypical = 9; and HR ASD = 11; unknown = 1. Complete case analysis is also reported.

Finally, to assess whether early measures of laterality could go on to predict later lateralised function such as motor ability and higher order cognitive abilities, the above measures at 6 months were used in multiple regression analyses to predict language and motor ability at 14 months as measured by the Mullen.

Based on previous literature we expected a main effect of Object for observations at both time points and a main effect of Side at 6 months and 14 months for saccade time. We expected a Side × Object interaction by 14 months and a Side × Object × Outcome interaction with the typical group being faster to look at faces on the left. We also expected looking left at faces to be positively associated with motor and language ability.

## Supplementary information

Supplementary file1 (DOCX 26 kb)
