# Author Correction: Gaze behaviour to lateral face stimuli in infants who do and do not receive an ASD diagnosis

**DOI:** 10.1038/s41598-020-72419-3

**Published:** 2020-09-24

**Authors:** Georgina Donati, Rachael Davis, Gillian S. Forrester

**Affiliations:** 1 Department of Psychological Sciences, School of Science, Birkbeck, University of London, Malet Street, London, WC1E 7HX UK; 2 Psychology Department, University of Edinburgh, Edinburgh, EC1V 0HB UK

Correction to: *Scientific Reports* 10.1038/s41598-020-69898-9, published online 06 August 2020

The original version of this Article contained errors.

In the title of the paper, the word “receive” was incorrectly given as “gain”.

In addition, in the Abstract,

“Cerebral lateralisation of function is common characteristic across vertebrate species and is positively associated with fitness of the organism, in humans we hypothesise that it is associated with *cognitive* fitness.”

now reads:

“Cerebral lateralisation of function is a common characteristic across vertebrate species and is positively associated with fitness of the organism, in humans we hypothesise that it is associated with *cognitive* fitness.”

In the Discussion,

“In term of observations, a marked difference in the rate of looking at faces vs looking at non-faces in all groups was found supporting previous literature showing that infants have a preference for looking at faces over other objects^49,50,51^”.

now reads:

“In terms of observations, a marked difference in the rate of looking at faces vs looking at non-faces in all groups was found supporting previous literature showing that infants have a preference for looking at faces over other objects^49,50,51^”

Finally, in the Methods section, subsection Measures,

“The Mullen Scales of Early Learning (MSEL) were assessed in the infants^55^.”

now reads:

“The Mullen Scales of Early Learning (MSEL) were assessed in the infants at 14 months^55^.”

These errors have now been corrected in the PDF and HTML versions of the Article, and in the accompanying Supplementary Information file.

