# Supplementary material for: Gaze behaviour to lateral face stimuli in infants who do and do not receive an ASD diagnosis
Source: Sci Rep. 2020 Aug 6;10:13185. doi: 10.1038/s41598-020-69898-9 (PMC7411063; doi:10.1038/s41598-020-69898-9)
Supplement: Supplementary file 1 — Supplementary file1 (DOCX 26 kb) [file 41598_2020_69898_MOESM1_ESM.docx]

**Supplementary Information: Gaze behaviour to lateral face stimuli in infants who do and do not receive an ASD diagnosis**

Georgina Donati^a^, Rachael Davis^b^ and Gillian S. Forrester^a^

^a^Department of Psychological Sciences, Birkbeck, University of London, Malet Street, London, W1CE 7HX

^b^Psychology Department, University of Edinburgh EC1V 0HB

Supplementary Note 1: Cohort Details

At the time of enrolment, none of the infants had been diagnosed with any medical or developmental condition. Infants in the at-risk groups all had an older sibling (male =45, female =9) with a clinical diagnosis of ASD and confirmed by two expert clinicians (PB, TC) based on information using the Development and Wellbeing Assessment (DAWBA) [1] and the parent-report Social Communication Questionnaire (SCQ) [2]. Parent reported family medical histories were examined for significant medical conditions in the proband or extended family members, with no exclusions made on this basis.

Infants in the low-risk group were recruited from the volunteer database at the Birkbeck Centre for Brain and Cognitive Development. Inclusion criteria included full-term birth (with one exception), normal birth weight, and lack of any ASD within first-degree family members (as confirmed through parent interview regarding family medical history). All low-risk infants had at least one older-sibling (in 3 cases, only half-sibling/s, male =28, female=22). Screening for possible ASD in these older siblings was undertaken using the SCQ.

The outcome groups in the at-risk cohort were characterised at 36-months as: those who were typically developing, those classified as having ASD, and those exhibiting some form of developmental concerns. ASD (including childhood autism; atypical autism, other pervasive developmental disorder (PDD)) was diagnosed using all available information from all visits by experienced researchers. From the 53 toddlers assessed at 36-months, 17 (11 boys, 6 girls) met criteria for an ASD diagnosis (32.1%). Given the young age of the children, and in line with the proposed changes to DSM-5 [3], no attempt was made to assign specific sub-categories of PDD/ASD diagnosis. Another subgroup of toddlers from the at-risk group (22.6%; 3 boys, 9 girls) who were classified as not having ASD but either scored above the ADOS or ADI [4] cut-off for ASD or scored <1.5SD on the Mullen ELC or RL and EL subscales but did not meet ICD-10 criteria for an ASD (9 scored > ADOS cut-off, 1 > ADOS cut-off and <1.5SD Mullen ELC cut-off, 1 > ADI cut-off, and 1 < 1.5SD Mullen ELC cut-off).

Supplementary References

[1] Goodman R, Ford T, Richards H, Gatward R, Meltzer H. The development and well-being assessment: description and initial validation of an integrated assessment of child and adolescent psychopathology. Journal of Child Psychology and Psychiatry 2000;41(5):645–55.

[2] Rutter M, Bailey A, Lord C. Social Communication Questionnaire-WPS SCQWPS. Los Angeles, CA: Western Psychological Services; 2003.

[3] American Psychiatric Association (APA). Diagnostic and Statistical Manual. 5^th^ ed., text rev. Washington, DC: American Psychiatric Association; 2012.

[4] ADI-R Lord C, Rutter M, Le Couteur A. Autism diagnostic interview-revised: a revised version of a diagnostic interview for caregivers of individuals with possible pervasive developmental disorders. Journal of Autism and Developmental Disorders 1994;24:659–85.

Supplementary Table 1: Regression Results:

|  | Observation Scores at 6months | | |  |  |
| --- | --- | --- | --- | --- | --- |
| Fine motor 14m |  | *ß* | *SE* | *t* | *p* |
|  | (Intercept) | 0.003 | 0.099 | 0.027 | 0.979 |
|  | Right Non-Face | -0.174 | 0.112 | -1.554 | 0.123 |
|  | Left Non-Face | -0.044 | 0.114 | -0.392 | 0.696 |
|  | Right Face | 0.113 | 0.113 | 1.001 | 0.319 |
|  | Left Face | 0.116 | 0.115 | 1.003 | 0.318 |
| Gross Motor 14m |  |  |  |  |  |
|  | (Intercept) | -0.010 | 0.099 | -0.098 | 0.922 |
|  | Right Non-Face | -0.007 | 0.110 | -0.061 | 0.952 |
|  | Left Non-Face | -0.192 | 0.113 | -1.698 | 0.093 |
|  | Right Face | -0.029 | 0.113 | -0.254 | 0.800 |
|  | **Left Face** | **0.259** | **0.115** | **2.258** | **0.026** |
| Receptive Language 14m |  |  |  |  |  |
|  | (Intercept) | 0.060 | 0.097 | 0.164 | 0.8701 |
|  | **Right Non-Face** | **-0.267** | **0.109** | **-2.445** | **0.016** |
|  | Left Non-Face | 0.090 | 0.112 | 0.806 | 0.422 |
|  | Right Face | 0.076 | 0.111 | 0.686 | 0.494 |
|  | Left Face | 0.037 | 0.113 | 0.323 | 0.747 |
| Expressive Language 14m |  |  |  |  |  |
|  | (Intercept) | 0.012 | 0.099 | 0.124 | 0.901 |
|  | **Right Non-Face** | **-0.249** | **0.111** | **-2.240** | **0.027** |
|  | Left Non-Face | -0.020 | 0.114 | -0.173 | 0.863 |
|  | Right Face | 0.049 | 0.113 | 0.436 | 0.664 |
|  | Left Face | 0.006 | 0.115 | -0.048 | 0.962 |
|  |  |  |  |  |  |
|  | Mean Saccade Scores 6 months |  |  |  |  |
| Fine motor 14m |  | *ß* | *SE* | *t* | *p* |
|  | (Intercept) | 0.015 | 0.133 | 0.116 | 0.908 |
|  | Right Non-Face | -0.084 | 0.168 | -0.499 | 0.621 |
|  | Left Non-Face | 0.144 | 0.141 | 1.021 | 0.314 |
|  | Right Face | -0.240 | 0.145 | -1.652 | 0.105 |
|  | Left Face | -0.095 | 0.142 | -0.669 | 0.508 |
| Gross Motor 14m |  |  |  |  |  |
|  | (Intercept) | 0.016 | 0.128 | 0.128 | 0.899 |
|  | Right Non-Face | -0.148 | 0.213 | -0.696 | 0.497 |
|  | Left Non-Face | -0.270 | 0.142 | -1.903 | 0.066 |
|  | Right Face | -0.082 | 0.149 | -0.549 | 0.586 |
|  | Left Face | -0.192 | 0.137 | -1.393 | 0.172 |
| Receptive Language 14m |  |  |  |  |  |
|  | (Intercept) | 0.002 | 0.133 | 0.015 | 0.988 |
|  | Right Non-Face | 0.025 | 0.184 | 0.135 | 0.894 |
|  | Left Non-Face | 0.082 | 0.142 | 0.579 | 0.566 |
|  | Right Face | -0.199 | 0.150 | -1.329 | 0.191 |
|  | Left Face | -0.139 | 0.131 | -1.063 | 0.293 |
| Expressive Language 14m |  |  |  |  |  |
|  | (Intercept) | -0.002 | 0.136 | -0.015 | 0.988 |
|  | Right Non-Face | -0.009 | 0.197 | -0.048 | 0.962 |
|  | Left Non-Face | -0.023 | 0.148 | -0.153 | 0.879 |
|  | Right Face | -0.061 | 0.161 | -0.382 | 0.704 |
|  | Left Face | -0.063 | 0.135 | -0.466 | 0.643 |
